# Diagnosis and Management of Pancreatic Cysts: A Comprehensive Review of the Literature

**DOI:** 10.3390/diagnostics13030550

**Published:** 2023-02-02

**Authors:** Ritu R. Singh, Harishankar Gopakumar, Neil R. Sharma

**Affiliations:** 1 Parkview Cancer Institute (PCI), Parkview Regional Medical Center, Fort Wayne, IN 46845, USA; 2 Department of Medicine, Indiana University School of Medicine, Fort Wayne, IN 46805, USA; 3 Division of Gastroenterology and Hepatology, University of Illinois College of Medicine, Peoria, IL 61656, USA

**Keywords:** pancreatic cystic lesions, endoscopic ultrasound, fine needle aspiration, surveillance, intraductal papillary mucinous neoplasm, mucinous cyst

## Abstract

The prevalence of pancreatic cysts has been rising due to the widespread use of cross-sectional imaging (CT scan and MRI) of the abdomen. While most pancreatic cysts are benign and do not require treatment or surveillance, a significant minority are premalignant and rarely malignant. The risk stratification of these lesions is not straightforward, and individual risk assessment, cyst size, distribution, and alarming morphologic features (when present) can guide the next steps in management. Neoplastic pancreatic cysts are mucinous or non-mucinous. Endoscopic ultrasound with fine-needle aspiration is often required to classify pancreatic cysts into mucinous and non-mucinous cysts and to assess the malignant potential. Advances in endoscopic techniques (confocal laser endomicroscopy, microforceps biopsy) can provide a definitive diagnosis of pancreatic cysts in some cases; however, the use of these techniques involves a higher risk of adverse events.

## 1. Introduction

Pancreatic cysts (PC) are the only known precursor lesions for pancreatic adenocarcinoma; however, only some of them are premalignant. While most PCs are detected incidentally on cross-sectional imaging, these modalities cannot completely risk-stratifying PCs on their own. The increasing use of CT scans and MRIs of the abdomen has resulted in a steady rise in the incidence of PC. The prevalence of PC varies widely and ranges from 3% to 20% depending on the imaging modality and population studied, with a higher prevalence in older adults undergoing MRI compared with the CT scan [1,2,3]. There is no correlation between increasing PC detection and pancreatic cancer incidence. This suggests that the rise in PC incidence is only the result of the increased resolution and utilization of abdominal imaging. PCs are not always benign, and there is a 5–8% risk of malignant transformation over 5- to 10 years of follow-up [4]. Thus, the discovery of a pancreatic cyst imposes a clinical surveillance and treatment dilemma along with tremendous stress on the patient and family.

A critical question following the detection of a PC is whether to treat, continue surveillance, or reassure the patient that it is benign. The answer has not been straightforward to date. However, improvements in the identification of high-risk features and the classification of PCs through non-invasive or minimally invasive techniques can ease ambiguity in this common imaging finding. Broadly, mucinous cysts are neoplastic and have higher malignant potential, and the first step of risk stratification has to focus on differentiating mucinous from non-mucinous cysts. Unfortunately, ongoing efforts to find a diagnostic test to reliably discriminate mucinous and non-mucinous or neoplastic and non-neoplastic benign cysts have been disappointing.

## 2. Classification of Pancreatic Cysts

PC can be broadly classified as neoplastic and non-neoplastic. Neoplastic PC can be mucinous or non-mucinous. Intraductal papillary mucinous neoplasms (IPMN) and mucinous cystic neoplasms (MCN) are the mucinous cysts. Most of the non-mucinous cysts, besides serous cystadenomas (SCA), arise from solid pancreatic lesions due to cystic degeneration and are either solid pseudopapillary neoplasms (SPN) or cystic pancreatic neuroendocrine tumors.

Not all PCs share similar malignant potential. Mucinous cysts (IPMN and MCN) have higher malignant potential depending on the degree of dysplasia [4]. SPNs are technically low-grade neoplasms [5,6]. SCAs are non-mucinous and have an extremely low risk of malignant transformation, and can be classified as non-neoplastic [7]. Pancreatic adenocarcinoma can degenerate into a cystic pancreatic lesion. Cystic degeneration is extremely rare (<1%) and is associated with poor prognosis [7]. However, there are also reports that cystic features are not rare in pancreatic carcinoma, and cystic changes are usually seen in poorly differentiated or undifferentiated tumors [8].

**Figure 1:**
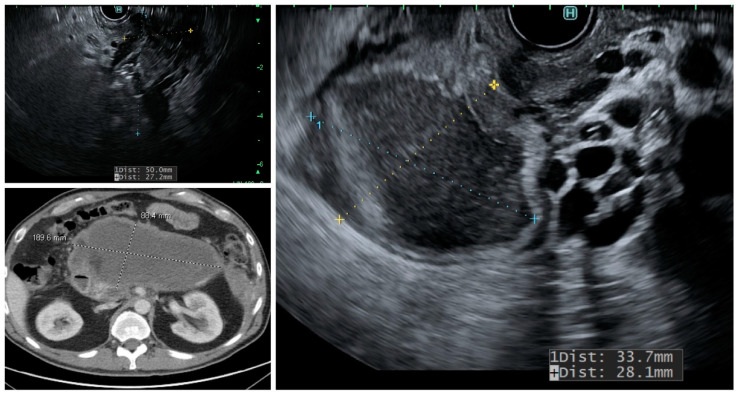
Endoscopic ultrasound and CT scan images showing pancreatic pseudocyst near the uncinate process.

The above classifications are based on the pathologic examination of the resected PC. Imaging modalities and the analysis of PC fluid can provide a clue towards identifying cyst types; however, the degree of dysplasia and its malignant potential can still be uncertain. Most non-neoplastic pancreatic cysts are pseudocysts (Figure 1) and are seen following acute pancreatitis or are associated with chronic pancreatitis.

### 2.1. Clinical and Demographic Features

SCAs are almost exclusively found in individuals younger than 55 and are mostly women. Other cyst types commonly seen in women are MCN and SPN. Additional clinical and imaging features can hint towards one of the three cyst types commonly seen in women. Patients with SCA rarely have jaundice or weight loss, and abdominal pain is uncommon. Jaundice and weight loss are also not seen in patients with SPN. Patients who harbor MCN are younger than 75-year females with a single PC. IPMN is commonly seen in patients older than 70 years and rarely less than 65 (Table 1).

#### Distribution and Morphologic Characteristics

**Figure 2:**
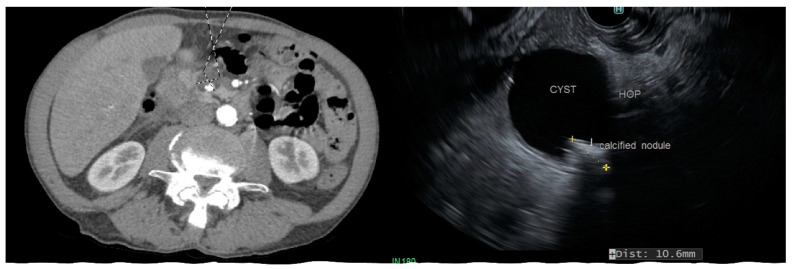
CT scan and endoscopic ultrasound demonstrating a side-branch IPMN involving the head of pancreas (HOP) with a calcified mural nodule.

**Figure 3:**
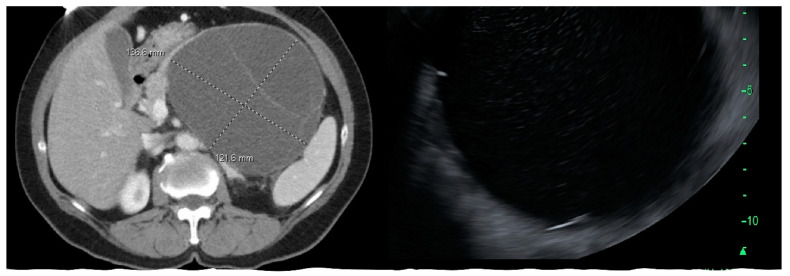
CT scan and endoscopic ultrasound images of mucinous cystic neoplasm in the body and tail of pancreas.

MCN, SCA, and SPN are solitary cysts. MCN and SCA lack communication with the main pancreatic duct and do not show pancreatic duct dilation on imaging. IPMN, by definition, demonstrate communication with the pancreatic duct, have the dilation of the duct, and are commonly multiple. IPMNs are more common in the head and neck region but can be found in other locations (Figure 2). They can be multifocal and should be considered along with pseudocysts when multiple cysts occur in the pancreas. MCNs are exclusively seen in the body or tail of the pancreas (Figure 3). While SCA can occur in the head, most SCA are located in the body or tail (~70–75%). There is no specific location of SPN (Table 1).

**Figure 4:**
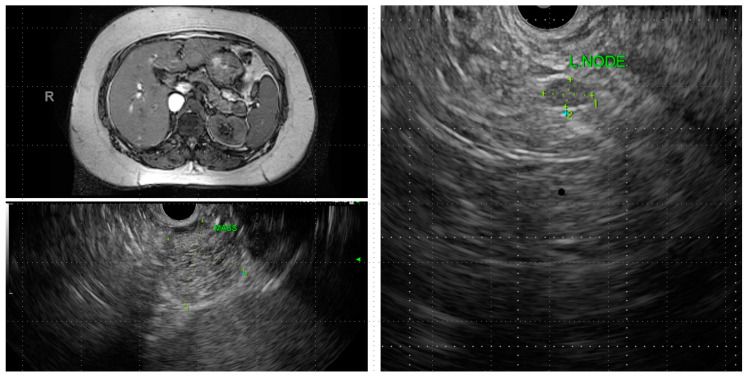
MRI and endoscopic ultrasound demonstrating a solid pseudopapillary neoplasm involving pancreatic tail with a regional lymph node involvement.

Cyst morphology can provide some insights into the type when characteristic features are present. SPN are characterized by the presence of solid components in the form of a mural nodule. A noticeable feature of SCA, found in about a quarter of cases, is the presence of calcification within the cyst, and this is distinct from the peripheral calcification that can be seen in MCN and SPN (Figure 4). This is sometimes referred to as a “central scar.” Side-branch and mixed-type IPMN are multiloculated and appear as clusters, while main-duct IPMN usually does not demonstrate a prominent cyst but are seen on imaging as a diffuse dilation of the main pancreatic duct.

### 2.2. Malignant Potential of Neoplastic Pancreatic Cysts

The risk of malignant transformation depends on the type and histology, the size of the lesion, the presence of solid components, the distribution of PC, and the age of the patient.

IPMN: The distribution of cysts in relation to the main pancreatic duct determines the malignant behavior of IPMN. Consequently, IPMN are classified as the main-duct IPMN (MD-IPMN) when MPD is dilated due to the direct involvement for more than 5 mm without a visible cyst, branch-duct IPMN (BD-IPMN), when there is a cyst, >5 mm without dilated MPD, and mixed-type when there is one or more PC > 5 mm along with the involvement of MPD. MD-IPMN and mixed-type IPMN have a higher risk of harboring invasive adenocarcinoma (approximately 45%) and high-grade dysplasia (approximately 60%), while BD-IPMN are at lower risk (16–20%) [11]. Moreover, the rate of cyst growth on follow-up can predict the risk of malignant behavior in BD-IPMN [12]. MPD dilation >10 mm in MD-IPMN, with a cyst diameter >40 mm in BD-IPMN, and the presence of a large mural nodule on EUS favor malignancy (Figure 2) [13]. Pergolini et al. followed up patients with BD-IPMN for over 10 years and found that the risk of malignancy was significant (approximately 8%) for cysts > 1.5 cm even after the five years of suggested surveillance period [14].

MCN: All MCNs are associated with a significant risk of high-grade dysplasia and invasive cystadenocarcinoma. The risk for high-grade dysplasia ranges from 6% to 13%, and for invasive adenocarcinoma, 4–12% [15,16]. The malignant risk increases with the increasing size of the lesion and the presence of the nodule [15]. Given the relatively younger age of onset, this risk justifies the surgical resection of the lesions unless constrained by advanced age, poor functional status, or comorbidities.

SPN: SPN are low-grade malignant neoplasms and usually run an indolent course. However, they carry the risk of metastatic spread, and liver and peritoneal deposits may be seen in 5–15% of cases (Figure 4) [17]. SPN is often diagnosed in young adults and are commonly present with an abdominal mass or abdominal pain, and are less commonly asymptomatic [18]. They are often well encapsulated and can potentially be cured with resection. Survival following surgical resection is non-inferior to people without SPN.

SCA: Serous cystic neoplasms carry an extremely low risk of malignant transformation [19]. A small subset (up to 5%) was found to have an aggressive tumor, and the risk of aggressive behavior correlates with the tumor size at diagnosis and location in the head of the pancreas [20]. Surgical resection should be considered in symptomatic patients with SCA and in cases where malignancy could not be excluded, especially those with oligocystic type [17,21]. For asymptomatic patients with SCA, with a low risk of malignancy, there is a lack of consensus on treatment. Surgically fit patients can be considered for follow-up; however, the tumor growth rate in the first few years is slow (~0.1 cm/year) [21].

## 3. Imaging and Image-Assisted Modalities in Diagnosis and Classification of PC

### 3.1. Cross-Sectional Imaging: CT Scan and MRI

A CT scan is the most widely obtained imaging modality for the evaluation of abdominal pathology in the emergency department and inpatient setting, and thus, the majority of PCs are discovered on abdominal CT performed for unrelated reasons. Contrast-enhanced pancreas protocol dual-phase or triphasic CT should be considered for the evaluation of PC. MRI with MRCP can demonstrate the communication of the cyst with MPD. Thus, it has better sensitivity and specificity at differentiating IPMN from other cysts (>90%) compared to a CT scan which has a sensitivity and specificity of less than 80% [22,23]. However, other studies have demonstrated the similar sensitivity of MRCP and multidetector CT in detecting communication between the cyst and pancreatic duct [24,25]. MRI also has a better performance at differentiating mucinous from non-mucinous cysts [22].

CT and MRI have a comparable sensitivity at differentiating malignant from benign PC, and both have equivalent accuracy at making a specific diagnosis (50–60%); however, the rate of misdiagnosis is remarkable with either imaging modality (>50%) [25,26,27]. MRI is considered better than CT at evaluating cyst morphology. Consequently, the major advantages of MRI and MRCP over CT scans are characterizing the aggressiveness of small cysts and detecting high-risk morphologic features, such as the mural nodule, septal thickening, and cyst communication with MPD (Figure 2).

### 3.2. Endoscopic Ultrasound (EUS)

**Figure 5:**
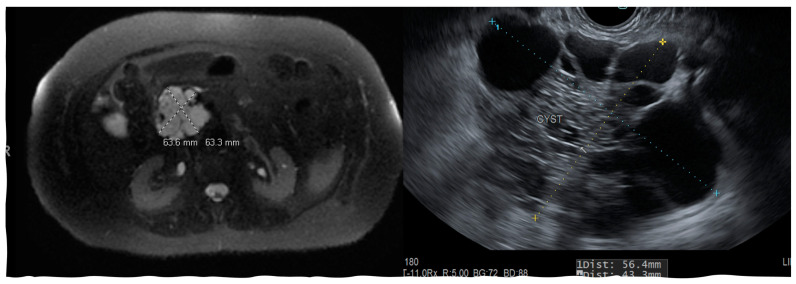
MRI and endoscopic ultrasound demonstrating a serous cystadenoma occupying the head and neck of pancreas.

EUS is considered the gold standard for pancreatic imaging. It produces high-resolution images of PC and provides samples for cytology and biochemical analysis through fine needle aspiration (FNA). In addition to the ability to aspirate cyst fluid, EUS can detect communication with pancreatic ducts and mural nodules and can demonstrate the characteristic images of SCA (Figure 5) [28,29]. The overall diagnostic accuracy of EUS morphology at differentiating mucinous from non-mucinous cysts is operator dependent, and even the experienced endosonographers have a modest agreement at differentiating neoplastic from non-neoplastic cysts (*K* = 0.24) [28,30]. However, diagnostic accuracy is greatly increased if the cyst is large enough to perform FNA.

In a large retrospective study comprising over 150 patients, Khasab et al. demonstrated the improved sensitivity of EUS (with or without FNA) in detecting neoplastic cysts and malignant cysts. There was an incremental yield of EUS over CT scans and MRI (36% to 54%) [31]. The sensitivity of MRI and EUS at the detection of mural nodules, MPD dilation, and communication with MPD may be comparable [32]. While EUS is routinely performed in PC >3 cm, smaller cysts can be neoplastic and harbor malignancy, while EUS can provide a clinical value when utilized in a variety of cysts [31].

Certain imaging characteristics favor the malignant potential of PC. MPD dilation ≥10 mm, a cyst or presence of an enhancing solid component are indicative of high-risk stigmata, and size >3 cm, septal wall thickening, dilated MPD 5–9 mm, a non-enhancing mural nodule, and peripancreatic lymphadenopathy suggest low-risk stigmata for malignant cysts, as per the international consensus guidelines for IPMN and MCN [33]. The presence of these features on cross-sectional imaging should prompt EUS-FNA for cytology and fluid analysis. FNA adds to the diagnostic performance of EUS by providing fluid for cytology and biochemical testing. Fluid cytology itself is diagnostic in half of the patients; however, the cyst fluid analysis of CEA, amylase, glucose, and genetic and molecular analysis add exponential value over cytology alone (Table 2) [28,31].

#### Contrast-Enhanced-EUS (CE-EUS)

The mural nodule has been shown to be an independent predictor of malignancy (invasive carcinoma or high-grade dysplasia) in IPMN [34]. CE-EUS involves real-time EUS imaging following the administration of an intravenous ultrasound contrast agent (microbubbles enclosed in a lipid shell). The use of a contrast agent facilitates the visualization of the microvasculature. CE-EUS is the best available imaging tool to characterize the mural nodule [29,34]. Lisotti et al., in a meta-analysis comprising over 500 patients, showed that CE-EUS has high sensitivity (88%) and specificity (79%) in detecting mural nodules within PC. Furthermore, in contrast, the harmonic mode of CE-EUS increased sensitivity to 97% and specificity to 90% for mural nodules [29,34].

### 3.3. Confocal Laser Endomicroscopy

Needle-based CLE (nCLE) utilizes a needle probe during EUS-FNA or fine needle biopsy (FNB) to obtain real-time images of the inner wall of the cyst. Three patterns on needle-based CLE (nCLE) differentiate most of the PC types with high specificity [35]. Mucinous cysts are characterized by epithelial features with papillae and epithelial bands, the trabecular pattern identifies cystic neuroendocrine lesions, and the fern pattern of vascularity is distinctive of SCA.

While a pilot study showed the low sensitivity (59%) of nCLE at differentiating PC types [36], subsequent studies have demonstrated optimal sensitivity arguing that there is a learning curve and the need to maintain proficiency and experience as an operator to apply nCLE to clinical practice. A multicenter, prospective validation study demonstrated high sensitivity, specificity, and accuracy (95%, 100%, and 97–99%, respectively) for the diagnosis of mucinous lesions and serous cystadenomas [37]. The accuracy of nCLE for mucinous cysts is significantly higher than cytology or CEA (97% vs. 71%), and nCLE distinguished the PC subtypes (SCA, cystic NEN, and SPN) with absolute precision (100% sensitivity, specificity, and accuracy) [38].

CLE is associated with a 3–9% risk of adverse events. Acute pancreatitis is the most common complication; other complications are intra-cystic bleeding, transient abdominal pain, and rarely cyst infection [36,37,38,39].

### 3.4. Microforceps Biopsy

A microforcep is introduced through the FNA needle to obtain a biopsy from the PC wall and mural nodule. Microforceps Biopsy (MFB) improves the diagnostic yield in combination with EUS-FNA. The rate of tissue acquisition with MFB is approximately 90%, with a diagnostic accuracy of 68–75% [40,41]. A systematic review (mostly including retrospective studies) comprising a pooled analysis of over 500 patients showed an improvement in the diagnostic yield (OR 4.79, *p* = 0.007) compared to FNA cytology [42]. In a retrospective analysis, the addition of MFB to nCLE led to the discontinuation of surveillance in 11% and avoidance of surgery in 25% of patients [41]. While the diagnostic yield of nCLE improved (by 93%) in combination with MFB and cytology, the improvement in the diagnostic yield was insignificant. This raises questions on the value of adding MFB in the setting of nCLE and FNA [41].

A concern with the use of MFB is the incidence of pancreatitis and other adverse events. A recent prospective study by Cho et al. showed a 7% (3 of 45 patients) adverse event rate [43]. A study by Facciorusso et al. focused on the predictors of adverse events in patients undergoing the EUS-guided needle biopsy of PC. They found that the use of these forceps was associated with a 28% risk of adverse events in IPMN and cautioned against the use of the cyst morphology was suspicious of IPMN [44]. A prospective single-center study including 101 patients who underwent EUS-guided through-the-needle biopsy reported adverse events in 10% of patients, with four demonstrating severe events and one fatality [45]. Thus, despite its high diagnostic yield, MFB raises significant safety concerns—particularly in use with potential IPMNs.

## 4. Cyst Fluid Analysis

### 4.1. Cytology

Morphologic features on EUS cannot reliably characterize PC [7]. While EUS-FNA with the cytology of PC improves the diagnostic accuracy of EUS, the sensitivity and negative predictive value are low. Two meta-analyses have shown a modest sensitivity of cytology (54% and 63%) for predicting mucinous cysts; however, the specificity ranged from 88 to 93% [46,47]. Likewise, the sensitivity of EUS-FNA cytology at differentiating benign and malignant IPMN has been shown to be low (65–75%). However, cytology is the most accurate at diagnosing malignant cysts [48,49].

### 4.2. Cyst Fluid Chemistry

Cyst fluid CEA has been shown to have good accuracy (86%) and decent sensitivity (81%) at differentiating mucinous from non-mucinous cysts using a cutoff of 192 ng/mL; however, it is unable to discriminate between malignant and benign cysts reliably [28]. A meta-analysis demonstrated a sensitivity of 63% and a specificity of 88% for CEA at differentiating mucinous from non-mucinous PC (Table 2) [46]. A large single-center study showed an accuracy of 86% with an area under the curve (receiver operating area) of 0.9 when using a cutoff of 109.9 ng/mL [50]. A retrospective study was conducted on 68 histologically confirmed PC-assessed CEA and CA-125 in the cyst fluid. The authors reported a sensitivity, specificity, and accuracy of 89%, 78%, and 84%, respectively, at differentiating mucinous from non-mucinous cysts using a CEA threshold of 67.3 ng/mL and differentiated by 78% of MCN from other cysts using a CA-125 threshold of 10 U/mL [51]. Conceivably, lowering the threshold of the cyst fluid CEA improves sensitivity but sacrifices specificity, and vice versa [46]. A lower threshold may be useful for predicting non-mucinous cysts in high-risk individuals.

Recent interest has risen in the estimation of cyst fluid glucose for differentiating mucinous and non-mucinous cysts due to its better performance, low cost, and ease of performance. Laboratory or point-of-care glucose using a cut-off of 40–50 mg/dL in PC fluid has a sensitivity of 88–95% and specificity of 78–92%, as demonstrated in retrospective studies with better accuracy than CEA [52,53]. A recent multicenter retrospective study comprising 93 patients found the excellent sensitivity (88%) and specificity (91%) of glucose in a fresh cyst fluid sample, and the area under the curve for glucose for diagnosing mucinous cysts was significantly higher compared with fluid CEA (0.96 vs. 0.81, *p* = 0.003) [54].

Fluid amylase <250 U/L can reliably exclude a pancreatic pseudocyst; however, its utility in identifying neoplastic PC is limited (Table 2) [55].

**Table 2:** Pancreatic cyst fluid analysis.

Cyst Fluid Analysis	Mucinous Cysts (MC) vs. Non-Mucinous Cysts	Solid Cystic Neoplasm	Pseudocyst	Benign vs. Malignant
Cytology	~Sn 60%			
Chemistry/tumor markers				
CEA	>192 ng/mL; Sn 50–77%, Sp 83–96%, accuracy 86% for MC vs. non-MC			
Glucose	<50 mg/dL Sn 88–95%, Sp 78–96%, accuracy 84–96% for MC vs. non-MC		Low glucose CEA improves diagnostic accuracy	
CEA + Glucose	CEA > 192 ng/mL and glucose <21 mg/dl Sn 93%, Sp 92%			
CA 19-9				<37 U/mL benign cysts
Amylase	Low		>250 U/L- high Sn and Sp	
VEGF-A [56]		>5000 pg/mL Sn 100% and Sp 84% In combination with CEA < 10 ng/mL Sn 96%, Sp 100%		
Next-generation sequencing				
*KRAS/GNAS*	*KRAS/GNAS* mutation Sn ~90%, Sp 100% for MC *Wildtype* gene *KRAS/GNAS* >90% accuracy for MC			
*TP53*, *PIK3CA* or *PTEN*	Improve Sn and Sp for MC vs. non-MC			Identification of advanced neoplasia Along with *KRAS/GNAS* mutation 80% Sn and 96% Sp for advanced neoplasia IPMN with advanced neoplasia
miRNA				Differential expression predicts advanced neoplasia in MC Malignant IPMN vs. adenoma Sn 80–90%, Sp 100%
Mucin				
*MUC5AC*, *MUC1*	*MUC5AC* > 90% accuracy detecting MC			*MUC1*-high-grade dysplasia
Sequential cyst fluid tests	Cytology/Mucin stain/CEA/String-sign Sn 96%, Sp 90%			

Sn—sensitivity, Sp—specificity, MC—mucinous cyst, IPMN—intraductal papillary mucinous neoplasm.

### 4.3. Molecular Analysis of Cyst Fluid

#### 4.3.1. DNA-Based: KRAS, GNAS

More recently, the next-generation sequencing of cell-free DNA from PC fluid obtained using EUS-FNA has gained some attention. Mucinous cysts, including both IPMN and MCN, commonly harbor mutations in the *KRAS* gene, and *GNAS* mutations are specifically seen in IPMN. *KRAS* and/or *GNAS* mutations are found in most histologically proven mucinous cysts with a sensitivity of 87–92% (Table 2) [57,58,59,60]. The detection of the wild-type *KRAS/GNAS* gene essentially excludes non-mucinous cysts (SPN, SCA) with a few exceptions (>90% confidence). *KRAS/GNAS* performed significantly superior to cytology (*p* = 0.001) and CEA (*p* = 0.02) in discriminating mucinous from non-mucinous cysts [58]. There was a good correlation between samples obtained surgically and through EUS-FNA [60].

The addition of *TP53*, *PIK3CA*, and *PTEN* can add to the identification of PC with advanced neoplasia (high-grade dysplasia or invasive adenocarcinoma). In a retrospective analysis, Rosenbaum et al. found the molecular analysis of DNA to be 100% specific for advanced neoplasia but with poor sensitivity (46%). However, the combination of cytology and non-*KRAS/GNAS* genetic variants increased sensitivity (76%) without sacrificing specificity (100%) [61]. Singhi et al. prospectively studied 626 PC fluid samples and found that mutation in *TP53*, *PIK3CA*, or *PTEN* along with *KRAS/GNAS* genes had a sensitivity of 79% with 96% specificity for advanced neoplasia in PC. Interestingly, the presence of mutant allele frequencies for alterations in *TP53*, *PIK3CA*, or *PTEN* genes that are at least equivalent to that of *KRAS/GNAS* mutant allele frequencies or the *GNAS* mutation with a mutant allele frequency of >55% increased the sensitivity (89%) and specificity (100%) for IPMN with advanced neoplasia [59].

#### 4.3.2. RNA-Based: Micro-RNA

Small non-coding segments of RNA that regulate gene expression are micro-RNA (miRNA). They have a potential role in oncogenesis and tumor progression, and they have been explored as molecular biomarkers for cancer diagnosis and prognosis. Several studies have assessed the role of miRNA in the detection of neoplastic PC. Differential expressions of miRNAs have been shown to predict advanced neoplasia in mucinous PC and differentiate malignant IPMN from adenomas with a sensitivity of 80–90% and specificity of up to 100% [62,63,64]. However, most of the studies have demonstrated different sets of miRNAs (miR-21/miR155, miR-24/miR-30a-3p/miR-18a/miR-92a/miR-342-3p/miR-99b/miR-106b/miR-142-3p/miR-532-3p, miR-711/miR-3679-5p/miR-6126/miR-6780b-5p/miR-6798-5p/miR-6879-5p) to detect malignant PC [62,63,64]. This limits the wide application of miRNA is used in the identification of neoplastic PC or diagnosing malignant IPMN. Thus, until a limited set of miRNAs that can reliably predict neoplastic PC is identified, its clinical use cannot be cost-effective and practical.

#### 4.3.3. Proteomics

Mucins are high molecular weight glycoproteins expressed by various epithelia and can be membrane-associated or secreted. Mucin has an important role in oncogenesis and tumor invasion, and the differential expression of mucin glycoforms can determine the biological aggressiveness of pancreatic neoplasms [65,66]. *MUC1* expression correlates with high-grade dysplasia in PanIN and pancreatic adenocarcinoma. *MUC5AC* expression is associated with most neoplastic pancreatic lesions and has good accuracy (91%) at detecting mucinous PC (sensitivity 78–89%, specificity 80–100%) (Table 2) [66,67,68]. However, it is unable to discriminate between types of PC. Given the differential expression of mucin glycoforms in neoplastic PC, the combined assessment of *MUC1*, *MUC2*, *MUC4*, and *MUC5AC* can be useful for classifying mucinous cysts and determining malignant risk [65].

## 5. High Risk Individuals (HRI)

Early detection is the key to curative resection, which is the goal of any cancer surveillance program. It is important to identify individuals who will benefit the most from a robust surveillance program. However, the overall low incidence of pancreatic cancer makes population-based screening impractical, given its costs and complexities [69,70]. The average lifetime risk of pancreatic cancer in the general population is about 1.3%, while HRIs are defined by a lifetime risk of >5% [71]. Age, family history, and germline mutations have been identified as the major factors that determine an increased risk of developing PC [72]. The specific surveillance recommendation is a combination of these risk factors, given the incomplete or low penetrance of pancreatic cancer susceptibility genes. Smoking is an independent risk factor for pancreatic cancer; however, in the absence of other risk features, smoking does not have definite HRIs. Moreover, there is no consensus on whether surveillance should be started at a younger age in an HRI with a smoking history [72].

### 5.1. Age of Presentation

In individuals with familial pancreatic cancer, the mean age at diagnosis is 68 years [71]. Studies have reported an increased incidence of pancreatic lesions in HRIs above 50 years of age and lesions with high-grade dysplasia above 65 years [73]. For individuals who meet the familial risk criterion without a defined genetic mutation, guidelines recommend beginning screening at the age of 50 or 10 years earlier than the age of diagnosis in a relevant family member [72,74]. Carriers of genetic mutations are considered at higher risk, and most societies recommend surveillance starting at age 50 [72,75]. Not all mutations confer the same risk, and hence earlier surveillance is suggested by some groups in higher-risk mutations such as CKDN2A (at 40 years) and Peutz-Jegher syndrome (30–40 years) [72].

### 5.2. Family History

The family history of pancreatic cancer, especially in first- and second-degree relatives, confers an increased lifetime risk of pancreatic cancer [73]. There is a 9-fold increased risk of developing pancreatic cancer in an individual with at least one first-degree relative (FDR) affected by pancreatic cancer. This risk increases with the number of affected FDRs, with up to 32-fold increased risk when there are three or more affected FDRs. Family history of non-pancreatic cancers (ovarian, colorectal, breast, and prostate) have also been observed to increase the risk of developing pancreatic cancer, although the evidence for these has been less favorable [76]. Family history has also been found to have a positive correlation with incidentally discovered pancreatic cysts with the risk of pancreatic cancer being higher in individuals with PCs and a family history of pancreatic cancer [77]. One of the limitations of family history is that pedigrees are often small, and there is a risk of incomplete information.

### 5.3. Germline Mutations

Several germline mutations have been associated with an increased lifetime risk of developing pancreatic cancer. About 10–20% of familial clustering of pancreatic cancers have been attributed to deleterious variants of pancreatic cancer susceptibility genes [78]. About 5–10% of apparently sporadic pancreatic cancers has also been attributed to these genes. Surveillance is recommended for carriers of deleterious variants of *BRCA2*, *BRCA1*, *MSH2*, *MLH1*, *PALB2*, *CDKN2A*, *STK11*, and *ATM* [72,73,74]. Given the high lifetime risk, surveillance is recommended in HRIs who are carriers of mutation or have CDKN2A and STK11, irrespective of a family history of pancreatic cancer. Screening is recommended for individuals with mutations in ATM, BRCA2, and PALB2 and if they also have a blood relative with pancreatic cancer. Individuals with hereditary pancreatitis associated with the pancreatitis susceptibility genes (PRSS1, CPA1, CPB2, and CTRC) have also been identified as HRIs, and surveillance for PC is recommended. In cases of CPA1 and CPB2 mutation associated with pancreatic cancer, the individuals do not have to progress through the clinical syndrome of pancreatitis. Surveillance recommendations for these are mostly based on expert consensus, and the recommendation is to start screening at age 40 or 20 years after the first pancreatitis attack [73].

## 6. Surveillance, Treatment or Reassurance

Following the discovery of an incidental pancreatic cyst, the consideration of a patient’s age and comorbidities is vital before embarking on extensive testing to determine the neoplastic potential of the PC. For patients with multiple other illnesses, an incidentally detected PC may not deserve ongoing attention. The Charlson comorbidity index is a useful tool in determining the probability of patient survival irrespective of the presence of PC and its progression to invasive cancer. Sahora et al. demonstrated an 11-fold higher probability of dying from causes unrelated to IPMN if the Charlson comorbidity index was more than six [79]. However, non-invasive imaging to define the size and morphologic features may facilitate discussions with the patient and family and provide them with relevant information to make the decision of further evaluation.

The focus of most society guidelines is to determine the indications for EUS/FNA, surgical considerations based on PC size, MPD dilatation, or the presence of solid components and surveillance [80]. A cyst size > 3 cm, MPD > 5–7 mm, change in MPD diameter, or solid cystic component on cross-sectional imaging are indications for EUS/FNA. In the absence of these features, a 1–2 yearly MRI for up to five years is recommended. Surgery is recommended for symptoms such as jaundice or acute pancreatitis that are attributable to PC, rapid cyst growth (>5 mm/year), the presence of a solid component within the cyst, mural nodule > 5 mm, or positive cytology on EUS/FNA. Furthermore, the American College of Gastroenterology (ACG) guidelines recommend surgical consideration of IPMN and MCN with new-onset diabetes, the obstructed or focal dilatation of MPD, or a cyst size > 3 cm. The American gastroenterology association and ACG guidelines recommend active MRI surveillance following the resection of a PC with advanced neoplasia for five years [80]. The European guidelines have a more conservative approach recommending the utilization of EUS/FNA or MRI every 6 months during the first year, then MRI or EUS annually. The European guidelines advocate for resection empirically based on size > 4 cm, symptomatic from the cyst or high-risk features [81].

## 7. Putting the Pieces Together

The diagnosis of pancreatic cysts and their management remains controversial. The guidelines are numerous and varied: the American Gastroenterology Association (AGA), Fukuoka, Sendai (International), European Society of Gastrointestinal Endoscopy (ESGE), International, and others have statements on cyst management. These guidelines were first released in 2006 through the international consensus group in Sendai, Japan [82]. Many societies have released updates to the guidelines; however, the most recent guideline updates are from 2018 (ACG and ESGE), and they struggle to incorporate the rapidly evolving technology which can improve cyst management.

While the guidelines themselves can provide some guidance, they must be tailored to the patient, and one must incorporate novel technology such as molecular markers, CLE, and even microforceps biopsies when appropriate. About 8–10% of pancreatic cysts that are resected in the US are benign. This number increases to 10–15% worldwide. These resections result in an avoidable cost of surgery and a potential increase in diabetes and other morbidities. On the other hand, the potential risk of an unresected pancreatic neoplasm is enormous—the 5-year survival for pancreatic cancer is 11% [83]. High-risk criteria in guidelines vary and can lead to unnecessary resections and missed carcinomas. For example, in a 2017 study looking at pancreatic resections for cysts, the AGA guideline would have avoided resection in 21 of 75 (28%) patients. However, 4 of 33 patients (12%) with high-grade dysplasia or malignancy would have missed the AGA guidelines, compared with none with the international association of pancreatology (IAP) or European guidelines [84].

Resection based on symptoms can help mitigate symptoms. It is important to delineate that the patient has abdominal symptoms related to recurrent pancreatitis and not another cause. Resection can lead to decreased recurrent acute pancreatitis if patients are carefully selected but do not change the risk of downstream cancer [85].

**Scheme 1:**
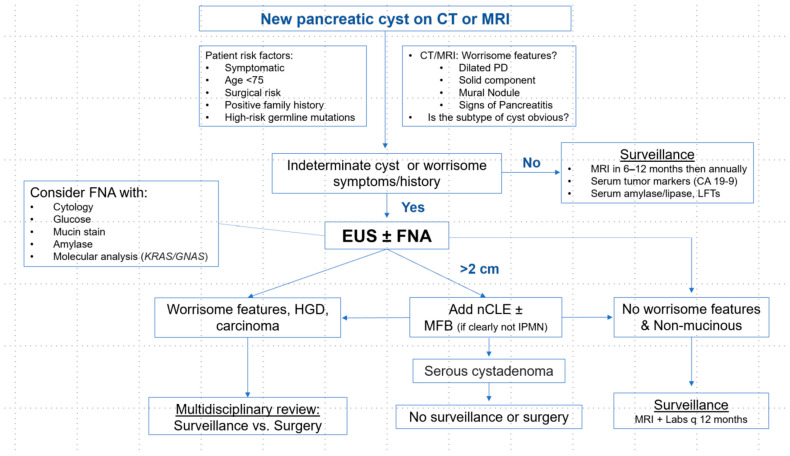
Flow diagram illustrating approach to a newly discovered pancreatic cyst. PD pancreatic duct, EUS endoscopic ultrasound, FNA fine-needle aspiration, HGD high-grade dysplasia, CLE confocal laser endomicroscopy, MFB microforceps biopsy, IPMN intraductal papillary mucinous neoplasm.

We recommend a combination of MRI with MRCP, serum labs (amylase, lipase, CEA, CA19-9, ALT, HbA1c), and the consideration of baseline EUS (with FNA if cyst >1 cm and CLE if cyst > 2 cm) in patients with an indeterminate cyst type, younger age (age < 75), positive family history, or germline mutation to better define the cyst subtype which can then ensure adequate surveillance. For patients undergoing FNA, we recommend obtaining cytology with mucin stain, glucose, and amylase and selectively testing for molecular analysis in suspected IPMN > 2 cm (Scheme 1). We reserve microforceps biopsies for use in cysts where the preceding labs, imaging, and EUS with FNA and CLE do not show evidence of mucinous cystic neoplasm to avoid the known complications from the use of MFB. From there, a cyst is often categorized into a “suspected subtype” based on the available information. An accurate diagnosis of the suspected subtype can help to avoid unnecessary surgeries.

This subtype can then have personalized surveillance based on patient history, age, and the characteristics of the cyst. The European guidelines (ESGE), while not perfect, may have the strongest level of evidence, and we encourage their use with a personalized approach after attempts to create and identify the cyst “suspected subtype.” We advocate for a multidisciplinary review and the use of repeat EUS with CLE, FNA, and molecular analysis prior to surgery for those who develop worrisome features during surveillance.

## 8. Conclusions

Pancreatic cyst management begins with an understanding of the patient: high risk vs. low risk depending upon the age of presentation, family history, and the presence of defined germline mutations. Then, imaging, labs, and EUS may help to define if the cyst is mucinous vs. non-mucinous and rule out the presence of malignancy. The utilization of imaging, labs, EUS with FNA, and novel technology such as CLE and molecular markers, in combination with understanding the presence of high-risk features, can allow for optimal surveillance to balance the risk of mortality from pancreatic cancer vs. cost and the burden of surveillance. The surveillance of pancreatic cysts continues to rapidly evolve, and recommendations from guidelines should be modified and tailored to the individual patient.

## Figures and Tables

**Table 1:** Clinical and demographic features of pancreatic cysts.

Cyst Type	Age of Presentation	Gender Predisposition	Clinical Presentation	Distribution	Morphologic Features	Malignant Potential
IPMN	>65–70 years [9,10]	M > F	1/3 of patients symptomatic (epigastric pain, back pain, weight loss), acute pancreatitis, new-onset diabetes, obstructive jaundice	Head and neck > body/tail	PD dilatation, BD- and mixed IPMN multiloculated	Depends on main PD involvement. MD-IPMN and mixed-IPMN malignant in 45–60%. High-risk: main PD > 1 cm, solid component or enhancing nodule, jaundice, HGD
Mucinous cyst	<70 years	F > M	Abdominal pain, weight loss, acute pancreatitis	Body or tail	Solitary, unilocular with ovarian-like stroma, peripheral calcification, no PD dilation	Malignant 4–12%; HGD 6–13%. High-risk: >6 cm, irregular thick wall, peripheral calcification
Serous cystadenoma	<55 years	F >> M	Rarely jaundice and weight loss	3/4 in body or tail	Solitary, “Central scar”, no PD dilation	Low malignant risk, ~5% aggressive
Solid pseudopapillary neoplasm	<30 years	F > M	Jaundice and weight loss uncommon	Any location, more commonly tail	Solitary, solid component, mural nodule, peripheral calcification	Low-grade malignant neoplasms, infrequently metastatic

IPMN Intraductal papillary mucinous neoplasms, M—male, F—female, PD—pancreatic duct, BD—branch duct, MD—main duct, HGD—high-grade dysplasia.

## Data Availability

Not applicable.
